# Association between sleep habits and symptoms of oral disease in adolescents: the 2017 Korea Youth Risk Behavior Web-based Survey

**DOI:** 10.1186/s12903-021-01575-3

**Published:** 2021-05-04

**Authors:** Eun-Sil Choi, Hyun-Sun Jeon, So-Jung Mun

**Affiliations:** 1 Department of Dental Hygiene, The Graduate School, Yonsei University, Wonju, Republic of Korea; 2 Department of Dental Hygiene, Yeoju Institute of Technology, Yeoju, Republic of Korea; 3 Department of Dental Hygiene, College of Software and Digital Healthcare Convergence, Yonsei University, 1 Yonseidae-gil, 26493 Wonju, Gangwondo Republic of Korea

**Keywords:** Oral health, Sleep, Adolescent, Quality of life

## Abstract

**Background:**

This cross-sectional study aimed to examine the relationship between sleep habits and oral disease symptoms in adolescents.

**Methods:**

Among 62,276 adolescents who participated in the 13th Korea Youth Risk Behavior Web-based Survey (2017), we selected a total of 54,766 adolescents (age, 12–18 years; male, 49.9%) for the final analysis, after excluding those who did not report their sleep duration. The 13th Korea Youth Risk Behavior Web-based Survey data were obtained from a stratified, multistage, clustered sample. Independent variables included general characteristics, oral health behavior, sleep types, sleep duration, and sleep quality; dependent variables comprised oral disease symptoms. Sleep was categorized according to bedtime astype A (bedtime < 1 a.m.) and type B (bedtime ≥ 1 a.m.). Data were analyzed using logistic regression analysis. Statistical significance was set at *p* < 0.05.

**Results:**

After adjusting for all covariates, adolescents with type A sleep had a higher risk of toothache on chewing (OR = 1.08, 95% CI 1.02–1.15) than adolescents with type B. Adolescents who slept for 6 h or less each night had a higher risk of pain in the tongue and buccal mucosa (OR = 1.35, 95% CI 1.18–1.54), gingival pain, and bleeding (OR = 1.31, 95% CI 1.19–1.45) than those who slept for more than 8 h. Adolescents with low quality of sleep had a higher risk of toothache or throbbing (OR = 1.70, 95% CI 1.60–1.81), toothache on chewing (OR = 1.73, 95% CI 1.65–1.82), and halitosis (OR = 1.51, 95% CI 1.41–1.59) than those with high quality of sleep.

**Conclusions:**

Our findings indicate that some oral symptoms are related to sleep duration and quality. It is essential to inculcate good sleeping habits in adolescents by emphasizing the effects of inadequate sleep duration and quality.

## Introduction

Sleep is an essential biological process in all humans [1] and an important factor in health and welfare [2]. Sleep duration less than 8 h or low sleep quality can have a negative impact on the psychological, physical, and social status of the individual. However, over the last 50 years, a steady decline in sleep duration has been observed due to erratic daily routine [3].

The National Sleep Foundation (USA) recommends 8–10 h of sleep for adolescents aged 14–17 years [4]. However, Statistics Korea’s data (2019) revealed that 63.2% of adolescents slept for less than 8 h, indicating that more than half of all adolescents in Korea sleep for a lesser duration than the recommended period [5]. Sleep duration of 8 h or more is an important factor in adolescents. It influences the emotional, behavioral, and cognitive aspects of development. It also creates a significant impact on school adjustment, interpersonal relationships, and learning outcomes [6]. Appropriate sleep habits are favorable for a healthy lifestyle and associated with sleep duration and sleep quality [7].

Studies have reported that sleeping habits established during adolescence persist in adulthood. Therefore, a healthy sleeping habit of more than 8 h is crucial for maintaining adolescent health and facilitating the transition to healthy adulthood [8, 9]. Adolescents must be made aware of healthy sleep habits, and their regular practice must be encouraged [10, 11].

Sleep duration can also affect oral health status. A study by Lee [12] reported a 2.28 times higher incidence of periodontal disease among adolescents who slept for less than 5 h than those who slept for more than 8 h. Moreover, shorter sleep duration in adolescents was associated with higher pain intensity in temporomandibular joint disorders, a typical musculoskeletal disorder of the orofacial region [13]. However, few studies on sleep and symptoms of oral disease in adolescents have been conducted.

In 2017, 60.1% of the Korean youth experienced oral disease symptoms, including tooth pain and bleeding, over 1 year [14]. Seirawan could not find a significant association between dental caries and school absenteeism, but students with tooth pain (a subjectively measured symptom) were 6 times more likely to be absent from school and receive 4 times lower than average scores than those without tooth pain [15]. Fewer subjective oral symptoms, including tooth or oral pain and bad breath, were associated with higher student well-being [16]. However, the rate of receiving oral health education in Korean youth in 2018 was low (27.6%), with inadequate reporting of living habits helpful for maintaining oral health [14].

According to the reviews on sleep worldwide, the bedtime for Asian adolescents is later than that for their peers in North America and Europe, with a decrease in total sleep duration and an increased rate of daytime sleepiness [17]. In the Wakayama Prefecture of Japan, students who slept late without absenteeism showed a higher risk of acquiring symptoms of headache, insomnia, depression, and anxiety [18]. According to a cohort study on 10-year-old children from Kuwait, the incidence of dental caries increased by 20% with lower levels of saliva leptin and higher ghrelin levels for every additional hour past 8 p.m. for bedtime. Thus, the sleep time of adolescents may have an impact on oral health. However, there have been few studies on the subject [19].

The present study assessed the association of type of sleep classified by adolescent bedtime with oral disease symptoms.

## Material and methods

### Research design and research tools

This cross-sectional study is a secondary analysis of descriptive research using data from the 13th Korea Youth Risk Behavior Web-based Survey (KYRBS) [20] to investigate the effect of sleep habits on oral disease experience among Korean youth. The raw data from the Korean Youth Risk Behavior Web-based Survey was approved before use by the Centers for Disease Control and Prevention (CDC) in Korea [21]. The study was conducted according to the relevant guidelines and regulations [20].

The KYRBS is a government-approved statistics survey (approval No. 117058) conducted every year since 2005 to identify health behaviors such as smoking, drinking, obesity, and eating habits among Korean youth. It is an anonymous self-filling web-based survey conducted for students from middle school to high school in Korea using a clustered, stratified, multistage probability sampling method [20].

### Subjects

A total of 54,776 out of 62,276 adolescents were selected, after excluding those who did not report the sleep duration details. The study participants were middle and high school students aged 12–18 years in Korean age (mean age: 15.0 years). During the KYRBS, each participant provided voluntary informed consent for participating in the survey [20].

### Description of variables

#### General characteristics

Gender, school levels, academic grades, and economic status were selected as analytical variables. Gender was divided into “male” and “female.” First-, second-, and third-year middle schoolers were reclassified as “middle school students.” First-, second-, and third-year high schoolers were classified as “high school students.” For classifications according to scholarly achievement and economic status over the most recent 12 months, “top,” “mid-upper,” “mid,” “middle-low,” and “low” were reclassified into “top,” “middle,” and “low” [20].

#### Oral health behavior

*Frequency of tooth brushing* The question asked was, “How many times did you brush your teeth yesterday?” A reply of “0” was excluded because there were no respondents, and only “1” and “2 or more” were classified.

*Flossing habit* The question asked was, “In addition to toothpaste and toothbrush, mark all the items you currently use for your oral health.” The answers were classified as “Used” and “Not used.” [20].

#### Sleep habits

*Sleep types* Sleep was divided into two types depending on individual bedtime. We classified early bedtime (before 1 a.m.) as type A and late bedtime (after 1 a.m.) as type B. This classification was based on a previous study [22] and the characteristics of our research data. *Sleep duration* Using the Korean Youth Risk Behavior Web-based Survey data, the sleeping period was discerned as a response to this question: “During the last 7 days, what time did you go to bed, and what time did you wake up from sleep?” The answers were to be written in hours and minutes separately for weekdays (Monday to Friday) and weekends (Saturday and Sunday). Sleep duration was divided into weekday and weekend in the raw data [20]. However, only “weekday” data were used in this study to help discern adolescent daily life. We calculated sleep duration based on the methodology followed in previous studies [23] and guidelines [20] for research data analysis. As the standard for adequate sleep for adolescents provided by the U.S. National Sleep Foundation is 8–10 h [4], the frequency distribution of sleep duration was classified as “less than 6 h,” “more than 6 h but less than 7 h,” “more than 7 h but less than 8 h,” or “more than 8 h.”

*Sleep quality* For the question, “Do you think that the amount of sleep you had over the past 7 days is sufficient for fatigue recovery” from the Korean Youth Risk Behavior Web-based Survey data, the responses “quite enough,” “enough,” “moderate,” “insufficient,” and “very insufficient” were reclassified into “enough,” “moderate,” and “insufficient” [20].

#### Experience of symptoms of oral disease

Symptoms related to oral disease were selected from the Korean Youth Risk Behavior Web-based Survey. Individuals were questioned about oral symptoms experienced during the last 12 months. Five items were used: “toothache or throbbing,” “toothache on chewing,” “gingival pain and bleeding,” “pain in the tongue and buccal mucosa,” and “halitosis” [20].

### Statistical analysis

Following the KYRBS data analysis guidelines, a complex sample data analysis was conducted using corrections for strata, cluster, weight, and a finite population. Using SPSS statistics-version 25.0, weights were applied to ensure appropriate representation of Korean youth in the web-based survey complex samples of youth risk behaviors [20].

Crossover analysis-logistic regression was used to examine the relation of symptom experience. For logistic regression analysis, adjusted variables were gender, school level, grade, economic status, tooth brushing frequency, flossing habits, sleep types, sleep duration, and sleep quality. Statistical significance was set at *p* < 0.05.

## Results

### General characteristics of the subjects depending on the type of sleep

Of the total 54,776 adolescents, 27,454 were males (50.1%) and 27,312 were females (49.9%). The average sleep duration for all subjects was 6 h 21 min. The number of adolescents who slept for less than 7 h was the highest at 33,997 (62.1%). The number of adolescents who responded that their sleep quality was poor was the highest at 23,239 (42.4%).

Type A had 18,350 middle school students (65.4%), which was more than the number of high school students. Type B had 18,562 high school students (69.5%), which was more than the number of middle school students. In type B, no one slept for more than 8 h.

In type A, the number of adolescents who responded that their quality of sleep was good was the highest at 10,637 (37.9%); in type B, the number of adolescents who responded that their quality of sleep was poor was the highest at 15,809 (59.2%). In type A, the average amount of sleep was 7 h 24 min (3 h 18 min–12 h max), and in type B, the average amount of sleep was 5 h 12 min (30 min–7 h 48 min max). Thus, the average amount of sleep was less in type B than in type A (Table 1).

**Table 1:** General characteristics, oral health behavior, sleep duration, sleep quality depending on sleep types among Korean youths (n = 54,766) : N(%)

Variables	Sleep types		Total
	A	B	
	28,052 (51.2)	26,714 (48.8)	54,766 (100.0)
Gender			
Male	15,394 (54.9)	11,918 (44.6)	27,312 (49.9)
Female	12,658 (45.1)	14,796 (55.4)	27,454 (50.1)
Mean age	14.4	15.7	15.0
School levels			
Middle	18,350 (65.4)	8152 (30.5)	26,502 (48.4)
High	9702 (34.6)	18,562 (69.5)	28,264 (51.6)
Academic grades			
High	11,212 (40.0)	10,510 (39.3)	21,722 (39.7)
Middle	8056 (28.7)	7722 (28.9)	15,778 (28.8)
Low	8784 (31.3)	8482 (31.8)	17,266 (31.5)
Economic status			
High	11,346 (40.4)	10,366 (38.8)	21,712 (39.6)
Middle	12,977 (46.3)	12,337 (46.2)	25,314 (46.2)
Low	3729 (13.3)	4011 (15.0)	7740 (14.1)
Frequency of tooth brushing^a^			
1	274 (1.0)	264 (1.0)	538 (1.0)
≥ 2	27,778 (99.0)	26,450 (99.0)	54,228 (99.0)
Flossing habits			
Used	3776 (13.5)	3293 (12.3)	7069 (12.9)
Not used	24,276 (86.5)	23,421 (87.7)	47,697 (87.1)
Sleep duration^b^			
< 6	1319 (4.7)	19,262 (72.1)	20,581 (37.6)
6 ≤ < 7	6634 (23.6)	6782 (25.4)	13,416 (24.5)
7 ≤ < 8	11,064 (39.4)	670 (2.5)	11,734 (21.4)
≥ 8	9035 (32.2)	0 (0.0)	9035 (16.5)
Sleep quality			
High	10,637 (37.9)	3147 (11.8)	13,784 (25.2)
Middle	9985 (35.6)	7758 (29.0)	17,743 (32.4)
Low	7430 (26.5)	15,809 (59.2)	23,239 (42.4)

^a^Times a day

^b^Hours

### The association between general characteristics, oral health behavior, sleep habits, and symptoms of oral disease

Type of sleep, sleep duration, and quality of sleep showed significant association with the experience of oral disease symptoms, such as “toothache or throbbing,” “toothache on chewing,” “gingival pain and bleeding,” “pain in the tongue and buccal mucosa,” and “halitosis” (*p* < 0.001) (Table 2).

**Table 2:** The associations between general characteristics, oral health behavior, and sleep habits with symptoms of oral diseases among Korean youths (n = 54,766) : N(%)

Symptoms	Toothache or throbbing	Toothache when eating	Gingival pain and bleeding	Pain in tongue and cheek inside mouth	Halitosis
Gender					
Male	5255 (19.6)	8850 (32.8)	4511 (16.6)	2521 (9.5)	6176 (22.6)
Female	7613 (28.1)	10,758 (39.5)	6095 (22.4)	3696 (13.9)	6024 (22.1)
* p*-value	< 0.001	< 0.001	< 0.001	< 0.001	0.218
School levels					
Middle	5400 (20.5)	8900 (34.0)	4733 (17.9)	2794 (10.9)	6000 (23.0)
High	7468 (26.3)	10,708 (37.8)	5873 (20.6)	3423 (12.2)	6200 (21.8)
*p-*value	< 0.001	< 0.001	< 0.001	< 0.001	0.004
Academic grades					
High	5012 (23.4)	7858 (36.3)	4228 (19.5)	2644 (12.5)	4580 (21.1)
Middle	3507 (22.5)	5378 (34.5)	2905 (18.5)	1685 (11.0)	3236 (20.6)
Low	4349 (25.4)	6372 (37.3)	3473 (20.2)	1888 (11.2)	4384 (25.4)
*p*-value	< 0.001	< 0.001	0.001	< 0.001	< 0.001
Economic status					
High	4572 (21.5)	7266 (33.9)	3985 (18.6)	2387 (11.3)	4136 (19.1)
Middle	5979 (23.8)	9002 (35.9)	4793 (18.9)	2762 (11.2)	5580 (22.1)
Low	2317 (30.0)	3340 (43.2)	1828 (23.8)	1068 (14.1)	2484 (32.4)
*p*-value	< 0.001	< 0.001	< 0.001	< 0.001	< 0.001
Frequency of tooth brushing^a^					
1	163 (30.6)	225 (42.8)	149 (29.8)	83 (16.0)	252 (47.2)
≥ 2	12,705 (23.7)	19,383 (36.0)	10,457 (19.4)	6134 (11.6)	11,948 (22.1)
*p*-value	< 0.001	0.001	< 0.001	0.001	< 0.001
Flossing habits					
Used	1644 (23.6)	2590 (37.1)	1482 (21.3)	871 (12.7)	1506 (21.0)
Not used	11,224 (23.8)	17,018 (35.9)	9124 (19.2)	5346 (11.5)	10,694 (22.5)
*p*-value	0.825	0.077	< 0.001	0.003	0.004
Sleep types					
A	5609 (20.2)	9015 (32.4)	4823 (17.3)	2644 (9.7)	5991 (21.5)
B	7259 (27.1)	10,593 (39.5)	5783 (21.5)	3573 (13.4)	6209 (23.1)
*p*-value	< 0.001	< 0.001	< 0.001	< 0.001	< 0.001
Sleep duration^b^					
< 6	5841 (28.2)	8288 (40.1)	4665 (22.4)	2887 (14.1)	4792 (23.1)
6 ≤ < 7	3180 (23.7)	4948 (36.9)	2599 (19.2)	1509 (11.5)	3054 (22.8)
7 ≤ < 8	2403 (20.5)	3857 (33.0)	2032 (17.4)	1126 (9.7)	2557 (21.8)
≥ 8	1444 (16.4)	2515 (28.3)	1310 (14.7)	695 (8.1)	1797 (20.2)
*p*-value	< 0.001	< 0.001	< 0.001	< 0.001	< 0.001
Sleep quality					
High	2227 (16.5)	3691 (27.1)	2059 (15.0)	1093 (8.2)	2518 (18.3)
Middle	3791 (21.5)	6071 (34.3)	3178 (17.9)	1756 (10.1)	3759 (21.2)
Low	6850 (29.6)	9846 (42.5)	5369 (23.1)	3368 (14.7)	5923 (25.4)
*p*-value	< 0.001	< 0.001	< 0.001	< 0.001	< 0.001

The data are determined from chi-square test (*p* < 0.05)

^a^Times a day

^b^Hours

### The association between sleep habits and oral symptoms

#### Toothache or throbbing

Adolescents who slept for 6 h or less had a 1.23-fold (OR = 1.23, 95% confidence interval [CI]  1.11–1.36) higher risk of toothache or throbbing of teeth than those who slept for more than 8 h. Adolescents with low quality of sleep had a 1.70-fold (OR = 1.70, 95% CI 1.60–1.81) higher risk of toothache or throbbing of teeth than those with high quality of sleep. No statistically significant difference was observed in the experience of toothache or throbbing symptoms for the type of sleep (Table 3).

**Table 3:** The association between sleep habits and oral symptoms among Korean youths (n = 54,766)

Variables	Toothache or throbbing	Toothache when chewing	Gingival pain and bleeding	Pain in tongue and buccal mucosa inside mouth	Halitosis
	Adjusted OR (95% CI)	Adjusted OR (95% CI)	Adjusted OR (95% CI)	Adjusted OR (95% CI)	Adjusted OR (95% CI)
Sleep types^a^					
A	1.00	1.00	1.00	1.00	1.00
B	1.06 (0.99–1.13)	1.08 (1.02–1.15)	0.99 (0.92–1.06)	1.06 (0.98–1.16)	1.00 (0.94–1.07)
Sleep duration^b^					
< 6	1.23 (1.11–1.36)	1.15 (1.06–1.26)	1.31 (1.19–1.45)	1.35 (1.18–1.54)	1.13 (1.02–1.24)
6 ≤ < 7	1.16 (1.07–1.26)	1.17 (1.09–1.25)	1.18 (1.08–1.29)	1.21 (1.09–1.36)	1.13 (1.04–1.22)
7 ≤ < 8	1.13 (1.05–1.22)	1.12 (1.05–1.19)	1.13 (1.04–1.22)	1.12 (1.01–1.24)	1.07 (1.00–1.14)
≥ 8	1.00	1.00	1.00	1.00	1.00
Sleep quality					
High	1.00	1.00	1.00	1.00	1.00
Middle	1.23 (1.16–1.30)	1.30 (1.24–1.37)	1.14 (1.07–1.21)	1.15 (1.06–1.25)	1.18 (1.11–1.25)
Low	1.70 (1.60–1.81)	1.73 (1.65–1.82)	1.43 (1.34–1.52)	1.60 (1.47–1.74)	1.51 (1.41–1.59)

The data were analyzed by enter logistic regression analysis

Adjusted for gender, school level, grade, household economic status, tooth brushing frequency, flossing habits

*OR* odds ratio, *CI* confidence interval

^a^Times a day

^b^Hours

#### Toothache on chewing

Adolescents with type A sleep had a 1.08-fold (OR = 1.08, 95% CI 1.02–1.15) higher risk of toothache on chewing than adolescents with type B. Adolescents who slept for 6 h or less had a 1.15-fold (OR = 1.15, 95% CI 1.06–1.26) higher risk of toothache on chewing than those who slept for more than 8 h. Adolescents with low sleep quality had a 1.73-fold (OR 1.73, 95% CI 1.65–1.82) higher risk of toothache on chewing than those with high quality of sleep (Table 3).

#### Gingival pain and bleeding

Adolescents who slept for 6 h or less had a 1.31-fold (OR = 1.31, 95% CI 1.19–1.45) higher risk of gingival pain and bleeding than those who slept for more than 8 h. Adolescents with low sleep quality had a 1.43-fold (OR = 1.43, 95% CI 1.34–1.52) higher risk of gingival pain and bleeding than those with high quality of sleep (Table 3).

#### Pain in the tongue and buccal mucosa

Adolescents with type B sleep had a 1.06-fold (OR = 1.06, 95% CI 0.98–1.16) higher risk of pain in the tongue and buccal mucosa than those with type A sleep. Adolescents who slept for 6 h or less had a 1.35-fold (OR = 1.35, 95% CI 1.18–1.54) higher risk of pain in the tongue and buccal mucosa than those who slept for more than 8 h. Adolescents with low sleep quality had a 1.60-fold (OR = 1.60, 95% CI 1.47–1.74) higher risk of pain in the tongue and buccal mucosa than those with high quality of sleep (Table 3).

#### Halitosis

Adolescents who slept for 6 h or less had a 1.13-fold (OR = 1.13, 95% CI 1.02–1.24) higher risk of halitosis than those who slept for more than 8 h. Adolescents with low quality of sleep had a 1.51-fold (OR = 1.51, 95% CI 1.41–1.59) higher risk of halitosis than those with high quality of sleep (Table 3).

## Discussion

This study aimed to identify the association of sleep types, sleep quality, and sleep duration with some oral disease symptoms in adolescents using the national representative data from the Korean Youth Risk Behavior Web-based Survey. After correcting all variables, regression analysis showed that the risk of the symptom “Toothache on chewing” was 1.08-fold higher in type A than in type B and 1.73-fold higher in adolescents who commonly experience low-quality sleep than high-quality sleep. The risk of the symptom “Pain in the tongue and buccal mucosa” was 1.35-fold higher in adolescents who sleep less than 6 h than those who sleep an adequate amount of more than 8 h.

Asaka et al*.* reported that children who sleep less than 8 h a day were 1.17 times more likely to have tooth decay [24] , while Lee showed that teenagers who sleep less than 5 h a day were 2.28 times more likely to have periodontal disease than those who sleep more than 8 h a day [12]. This study surveyed self-report of oral disease symptoms without assessing actual disease appearance. However, multiple studies have used self-reported oral symptoms as a reliable marker for actual disease appearance [25–27]. Therefore, it is plausible to suggest that the association between sleep duration and oral symptom experience (dental pain and hemorrhage) defined in this study is comparable to the literature results elaborated above. Alqaderi et al. [19] reported an increased incidence of tooth decay with a delay in the sleep onset time. This result was similar to the increased incidence of “Toothache on chewing” in the type B sleep defined in this study. However, direct comparison between this study and preexisting literature showed limited results because preexisting literature categorized research subjects into “children” and “adolescents,” with a limited number of studies conducted on “adolescents.”

Type B sleep appeared more than type A in high school students. In Korea, the amount of time spent at school depends on the school, but high school students start their days earlier than middle school students [28]. For adolescents, sleep duration or sleep quality may depend on school starting time [29]. Hence, delaying school starting time could address the problem of sleep duration and skipping breakfast [30, 31]. Therefore, it is necessary to investigate how school starting times in different countries are associated with bedtimes and oral health in adolescents. Previous studies reported that sleep habits were better in middle school students than in high school students. Further, short sleep duration correlated with improved grades [31] and increasing competitive pressure during university entrance exams [32, 33].

Inadequate sleep can cause stress [34, 35], depression [36], and immunity [37]. Stress may cause oral disease [38]. Several studies have reported a statistically significant relationship between periodontal disease and cortisol levels in saliva [39, 40], an indicator of stress [41]. The subjects included in the present study were adolescents highly susceptible to oral disease due to school-related stress. Proper sleep is necessary to cope with stress [35]. Therefore, sleep is an important aspect to be considered in preventing oral diseases in adolescents.

There is sufficient evidence to demonstrate the effect of fluoride toothpaste on preventing tooth decay [42]. The use of fluoride toothpaste is recommended for the prevention of tooth decay in Korea [43]. As of 2012, 70.2% of Korean adolescents aged 5–19 years used fluoride, usually fluoride toothpaste [44]. However, further analysis was not possible because the nationwide online teenager health behavior survey providing data for this research did not specifically collect data on the use of fluoride toothpaste.

In the present study, sleep habits were categorized based on the duration, quality, and type of sleep. However, the difference in oral symptoms based on sleep types was minimal. This finding indicates that sleep duration and quality are more related to the experience of oral disease symptoms than the type of sleep. Thus, it is important to ensure good sleep quality and duration in young individuals instead of following specific sleep patterns.

The data included in the present study were self-entry web-based surveys, and the subjects were instructed to fill in their subjective symptoms with a “yes” or “no” reply. Therefore, there is a possibility of bias in the information provided. However, we used stratified sampling to minimize sampling error, and the self-filling web-based survey method prevented deliberate/unintentional errors by researchers observed in offline research.

Understanding subjective perception is also important. Perceived symptoms of oral disease are related to the objective measure of oral health status [45]. In some instances, subjective perception may be more accurate than clinical assessment represented by used dental services [46]. Therefore, despite the limitations of the present study, the use of a self-filling web-based survey is justified.

The present study is associated with two positive aspects. First, the relationship between sleep habits and oral symptom experience was assessed using different parameters, including two types of sleep patterns, sleep duration, and quality of sleep. Second, data was used from the 13th KYRBS [20], a national data representing Korean youth.

The present study uses a cross-sectional design; therefore, assessment of causal relationships between sleep habits and the five oral disease symptoms is challenging. Therefore, a prospective cohort study should be conducted to clarify the causal and temporal relationship between sleep habits and the experience of oral disease symptoms. Reports suggest that improper sleeping habits cause daytime drowsiness. Future studies should also consider tooth fracture or TMJ disease associated with accidents caused due to daytime sleepiness and symptoms of head and neck diseases related to sleep. Furthermore, this study evaluated only two types of sleeping patterns, and further research should include more diverse sleep types.

## Conclusions

Sleep types, sleep duration, and sleep quality among Korean teens showed cross-sectional relationships with oral disease experience.

Sleep duration and sleep quality showed a significant association with oral disease symptoms than the type of sleep. It is necessary to create awareness of proper sleep habits among adolescents by emphasizing the importance of adequate sleep duration and quality than the sleep types.

## Data Availability

The data that support the findings of this study are available from the Korea Centers for Disease Control and Prevention. Restrictions apply to the availability of these data, which were used under license for this study. Data are available at http://www.cdc.go.kr/yhs/home.jsp with the permission of the Korea Centers for Disease Control and Prevention.

## Abbreviations

USA: United States of America
KYRBS: Korea Youth Risk Behavior Web‐based Survey
CDC: Centers for Disease Control and Prevention
TMJ: Temporomandibular joint
OR: Odds ratio
CI: Confidence interval

## References

[1] Aldabal L, Bahammam AS (2011). Metabolic, endocrine, and immune consequences of sleep deprivation. Open Respir Med J.

[2] Wiener RC (2016). Relationship of routine inadequate sleep duration and periodontitis in a nationally representative sample. Sleep Disord.

[3] Larcher S, Benhamou PY, Pepin JL, Borel AL (2015). Sleep habits and diabetes. Diabetes Metab.

[4] National Sleep Foundation. Teens and sleep. https://www.sleepfoundation.org/articles/teens-and-sleep. Accessed 10 March 2019.

[5] Statistics Korea. Survey on child and adolescent human rights: average sleep time. http://kosis.kr/statHtml/statHtml.do?orgId=402&tblId=DT_ES2017_031&conn_path=I2. Accessed 10 March 2019.

[6] Choi KI (2012). An effect of sleeping time on school adaptation of youths: mediated by depression and ability of self-protection. Forum Youth Cult.

[7] Brown FC, Buboltz WC, Soper B (2002). Relationship of sleep hygiene awareness, sleep hygiene practices, and sleep quality in university students. Behav Med.

[8] Dahl RE, Lewin DS (2002). Pathways to adolescent health sleep regulation and behavior. J Adolesc Health.

[9] Shochat T, Cohen-Zion M, Tzischinsky O (2014). Functional consequences of inadequate sleep in adolescents: a systematic review. Sleep Med Rev.

[10] Malone SK (2011). Early to bed, early to rise?: An exploration of adolescent sleep hygiene practices. J Sch Nurs.

[11] You MA, Kang NG, Lee HJ (2017). Relationships between sleep habits, daytime sleepiness and problem behaviors among adolescents. J Digit Converg.

[12] Lee SH (2017). Relationship of sleep duration to periodontal disease in youth. J Digit Converg.

[13] Kim BS, Heo JY, Ok SM, Kim KH, Jeong SH, Ko MY (2013). Cyber leisure activities and physical activities in adolescents with temporomandibular disorder. J Oral Med Pain.

[14] Ministry of Health and Welfare of Korea, Centers for Disease Control and Prevention, Ministry of Education. The 13th Korea Youth Risk Behavior Web-based Survey Statistics. Sejong, Korea: MHWK; 2017.

[15] Seirawan H, Faust S, Mulligan R (2012). The impact of oral health on the academic performance of disadvantaged children. Am J Public Health.

[16] Tuchtenhagen S, Bresolin CR, Tomazoni F, da Rosa GN, Del Fabro JP, Mendes FM (2015). The influence of normative and subjective oral health status on schoolchildren's happiness. BMC Oral Health.

[17] Gradisar M, Gardner G, Dohnt H (2011). Recent worldwide sleep patterns and problems during adolescence: a review and meta-analysis of age, region, and sleep. Sleep Med.

[18] Habukawa C, Nagamitsu S, Koyanagi K, Nishikii Y, Yanagimoto Y, Seiji Y (2020). Late bedtime reflects QTA30 anxiety symptoms in adolescents in a school checkup. Pediatr Int.

[19] Alqaderi H, Tavares M, Al-Mulla F, Al-Ozairi E, Coodson JM (2020). Late bedtime and dental caries incidence in Kuwaiti children: a longitudinal multilevel analysis. Community Dent Oral Epidemiol.

[20] Korea Centers for Disease Control and Prevention (2017). Raw data guidelines of the 13th Korea Youth Risk Behavior Web-based Survey 2017.

[21] Korea Centers for Disease Control and Prevention. Raw data of the 13th Korea Youth Risk Behavior Web-based Survey 2017. http://www.kdca.go.kr/yhs/. Accessed 20 February 2021.

[22] Merikanto I (2013). Late bedtimes weaken school performance and predispose adolescents to health hazards. Sleep Med.

[23] Kim YS, Kim SH, An HG (2015). Predictive study on factors affecting sleep hours in Korean adolescents: based on 2012 adolescent health behavior survey statistics online. JKDAS.

[24] Asaka Y, Sekine M, Yamada M, Tatsuse T, Sano M (2020). Association of short sleep duration and long media use with caries in school children. Pediatr Int.

[25] Park SY (2020). Convergence relationship of BMI, sleep time and experience of oral disease in adolescents. J Korea Converg Soc.

[26] Lee WJ, Choi BY, Hwang KG (2018). The effect of gender between the oral symptoms experience and health behavior factors. J Korean Soc Dent Hyg.

[27] Woo HS, Shim YS (2014). Correlation between high school students' experience of visiting the dental clinic and oral symptoms. J Korean Contents Assoc.

[28] Rhie S, Chae KY (2018). Effects of school time on sleep duration and sleepiness in adolescents. PLoS ONE.

[29] Wheaton AG, Chapman DP, Croft JB (2016). School start times, sleep, behavioral, health, and academic outcomes: a review of the literature. J Sch Health.

[30] Alfonsi V, Scarpelli S, D’Atri A, Stella G, De Gennaro L (2020). Later school start time: the impact of sleep on academic performance and health in the adolescent population. Int J Environ Res Public Health.

[31] You MA (2017). Relationships between sleep habits, daytime sleepiness and problem behaviors among adolescents. J Digit Converg.

[32] Wang H, Hu R, Du H, Fiona B, Zhong J, Yu M (2018). The relationship between sleep duration and obesity risk among school students: across—setional study in Zhejiang, China. Nutr Metab.

[33] Lee JJ, Kang JH, Lee SK, Chae KY (2013). Impact of Sleep Duration On Emotional Status In Adolescent. J Korean Child Neurol Soc.

[34] Chiang JJ, Cole SW, Bower JE, Irwin MR, Taylor SE, Arevalo J (2019). Daily interpersonal stress, sleep duration, and gene regulation during late adolescence. Psychoneuroendocrinology.

[35] Choi DW, Chun SY, Lee SA, Han KT, Park EC (2018). Association between sleep duration and perceived stress: salaried worker in circumstances of highworkload. Int J Environ Res Public Health.

[36] Zhai L, Zhang H, Zhang D (2015). Sleep duration and depression among adults: a meta-analysis of prospective studies. Depress Anxiety.

[37] Perez de Heredia F, Garaulet M, Gomez-Martinez S, Diaz LE, Warnberg J, Androutsos O (2014). Self-reported sleep duration, white blood cell counts and cytokine profiles in European adolescents: the HELENA study. Sleep Med.

[38] Rai B, Kaur J, Anand SC, Jacobs R (2011). Salivary stress markers, stress, and periodontitis: a pilot study. J Periodontol.

[39] Goyal S, Jajoo S, Nagappa G, Rao G (2011). Estimation of relationship between psychosocial stress and periodontal status using serum cortisol level: a clinico-biochemical study. Indian J Dent Res.

[40] Refulio Z, Rocafuerte M, de la Rosa M, Mendoza G, Chambrone L (2013). Association among stress, salivary cortisol levels, and chronic periodontitis. J Periodontal Implant Sci.

[41] Michels N, Sioen I, Huybrechts I, Bammann K, Vanaelst B, De Vriendt T, Iacoviello L, Konstabel K, Ahrens W, De Henauw S (2012). Negative life events, emotions and psychological difficulties as determinants of salivary cortisol in Belgian primary school children. Psychoneuroendocrinology.

[42] Walsh T, Worthington HV, Glenny AM, Marinho VC, Jeroncic A (2019). Fluoride toothpastes of different concentrations for preventing dental caries. Cochrane Database Syst Rev.

[43] Cho EB. The use of fluoride for the prevention of dental decay in Korea and Its Recommendation. Korea Health Promotion Institute. 2017. Weekly issue 29. https://www.khealth.or.kr/kps. Accessed 20 Feb 2021.

[44] Statistics Korea. How to use fluoride. https://kosis.kr/statHtml/statHtml.do?orgId=213&tblId=DT_21301_023_01&conn_path=I2. Accessed 20 Feb 2021.

[45] Lee JH, Kim BS (2015). Relationship between the objective oral health status and the subjective oral health awareness of Korean adult. Asia Pac J Multimed Serv Converg Art Humanit Sociol.

[46] Aday LA, Andersen R (1974). A framework for the study of access to medical care. Health Serv Res.

